# Preparation, Antibacterial Activity, and Catalytic Application of Magnetic Graphene Oxide‐Fucoidan in the Synthesis of 1,4‐Dihydropyridines and Polyhydroquinolines

**DOI:** 10.1002/open.202100221

**Published:** 2021-12-01

**Authors:** Aliakbar Nosrati, Sara Amirnejat, Shahrzad Javanshir

**Affiliations:** ^1^ Heterocyclic Chemistry Research Laboratory Chemistry Department Iran University of Science and Technology Tehran 16846-13114 Iran

**Keywords:** antibacterial activity, fucoidan, Hantzsch reaction, magnetic graphene oxide, polymer-coated nanoparticles

## Abstract

Polymer‐coated magnetic nanoparticles are emerging as a useful tool for a variety of applications, including catalysis. In the present study, fucoidan‐coated magnetic graphene oxide was synthesized using a natural sulfated polysaccharide. The prepared BaFe_12_O_19_@GO@Fu (Fu=fucoidan, GO=graphene oxide) was characterized using Fourier‐transform infrared (FTIR) spectroscopy, scanning electron microscopy (SEM), transmission electron microscopy (TEM), energy‐dispersive X‐ray (EDX) analysis, vibrating sample magnetometry (VSM), thermogravimetric analysis (TGA), Raman spectroscopy, and X‐ray diffraction (XRD). The catalytic proficiency of BaFe_12_O_19_@GO@Fu was investigated in the synthesis of 1,4‐dihydropyridine and polyhydroquinoline derivatives. Excellent turnover numbers (TON) and turnover frequencies (TOF) (6330 and 25320 h^−1^) testify to the high efficiency of the catalyst. Moreover, the antimicrobial activity of BaFe_12_O_19_@GO@Fu was evaluated against *Escherichia coli (E. coli)*, and *Staphylococcus aureus* (*S. aureus*) through the Agar well diffusion method, indicating that BaFe_12_O_19_@GO@Fu has antibacterial activity against *S. aureus*.

## Graphical abstract

Catalyst recyclability and reusability are important issues in terms of economic and environmental protection aspects. In both model reactions for the synthesis of dihydropyridines and polyhydroquinolines, the catalyst was separated from the reaction mixture by an external magnet, washed several times with hot ethanol, and followingly by dried in an oven to be ready for the next runs. The recycled catalyst can be reused more than six times with no significant decrease in the catalytic activity (Figure 9c).

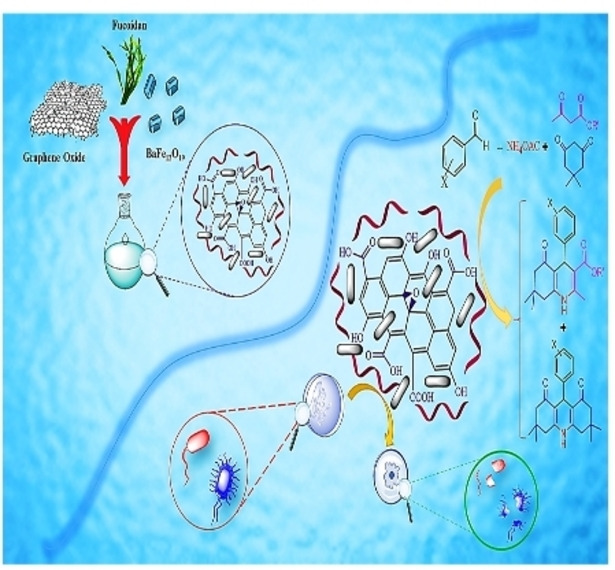

## 1 Introduction

Natural biopolymers have aroused renewed interest as an efficient tool in the development of biodegradable materials.[^1^, ^2^, ^3^] Their particularly attractive and desirable properties including nontoxic nature, biocompatibility, and biodegradability along with plentiful sources amongst natural materials make them prospective materials for many uses.^[4]^ Recently, carbohydrate‐based marine polymers have been widely used in the construction of nanocomposites, due to their high number of surface functionalities and biocompatibility, making them useful as heterogeneous catalysts.[^5^, ^6^, ^7^] Fucoidan (Fu), one of the most substantial cost‐effective marine polysaccharides originating from brown algae, is a fucose‐rich hygroscopic sulfated polysaccharide containing negative charges, due to its sulfate functional groups, which allow it to form complexes with other molecules of opposite charge.[^8^, ^9^] Moreover, Fu, built of a backbone of α(1→3)‐l‐fucopyranose residues having various substituents, has attracted great attention as capping material to design new nanocomposites. Besides, outstanding pharmaceutical applications of fucoidan including antibacterial, anticoagulant, immune, and anti‐thrombotic effects, have been widely studied and established.[^3^, ^10^, ^11^]

Nanocomposite technology is considered an effective strategy in materials research and development of polymer nanocomposites by using low‐loaded fillers, such as carbon nanomaterials (carbon nanotubes and graphene sheets), clay, hydroxyapatite, etc. Among these, graphene oxide (GO) with a remarkable amount of oxygenated groups such as hydroxyl, epoxide, and carboxyl groups provides effective means to alter the interactions between polymer chains and GO sheets and the reinforcement of nanocomposites.[^12^, ^13^, ^14^, ^15^] However, for the separation of GO, a high‐speed centrifuge is needed, which would discourage its application. Nevertheless, this disadvantage can be eliminated using magnetic GO which can be easily separated from the solution by a magnet.

During recent years, metal nanoparticles especially magnetic nanoparticles (MNPs) as a solid support material for the development of magnetically retrievable catalytic systems have attracted attention due to the easy separation, high surface area, accessibility, high level of chemical and thermal stability, and recoverability which have been considerably developed.[^16^, ^17^, ^18^, ^19^] Moreover, MNPs have gained increasing attention due to their promising applications of including biomolecular sensing, biomedical applications, pigments, and heterogeneous catalysis. Among them, barium hexaferrite (BaFe_12_O_19_), M‐type permanent magnet, showed promising applications in heterogeneous catalysis and microwave absorbing materials.[^20^, ^21^]

Considering the above‐mentioned facts, in this work, we have designed and developed a new magnetic heterogeneous catalyst using a natural sulfated polysaccharide, Fucoidan, for the functionalization of GO magnetized by barium ferrite named BaFe_12_O_19_@GO@Fu providing a unique combination of excellent properties.

Interesting features of this catalyst including the synergistic effect of Fucoidan with graphene oxide which improved its catalytic capacity in different conditions due to the presence of functional groups on the surface of both materials.^[22]^ In the other words, not only the negative charge of Fucoidan can catalyze the reactions demanding a basic catalyst.^[23]^ but also, the presence of graphene oxide because of carboxylic acid and proton donor groups on its surface enables to catalyze the reactions in which need to acidic catalysis, and besides the bi‐functional perspective of this part of the catalyst, thanks to the barium ferrite with noticeable magnetic properties make catalyst separation convenient at the end of the reaction. Finally, the high efficiency of this catalyst in the synthesis of 1,4‐dihydropyridines and polyhydroquinolines, is worth noting.

**Figure 1:**
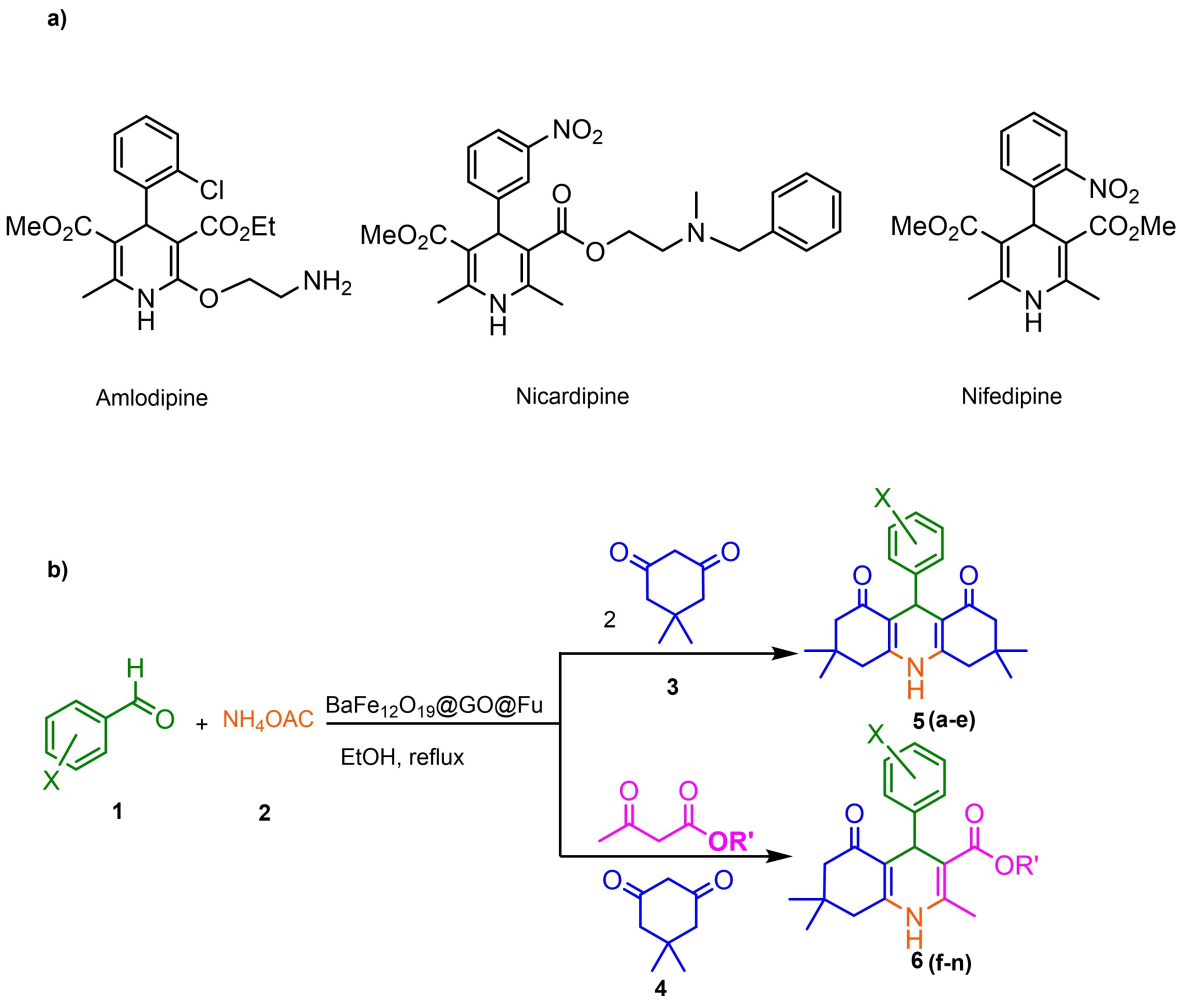
a) Examples of 1,4‐dihydropyridine pharmaceutical derivatives, b) BaFe_12_O_19_@GO@Fu catalyzed green synthesis of 1,4‐dihydropyridines **5 a**–**e** and polyhydroquinoline **6 f**–**n**

Nowadays, numerous structural analogs of these compounds are used in pharmacy and medicine.^[24]^ For instance, amlodipine, nicardipine, and nifedipine belong to the dihydropyridine family (Figure 1a).^[25]^ The classical approach for the synthesis of these compounds is one‐pot condensation reactions of various aldehydes with β‐ketoesters, dimedone, and ammonium acetate under drastic conditions. In recent decades, some improved procedures using an extensive range of catalysts including magnetic chitosan‐terephthaloyl‐creatine,^[26]^ BiFeO_3_,^[27]^ and γ‐Fe_2_O_3_/Cu@cellulose,^[28]^ Keggin‐type heteropolyacid H_5_BW_12_O_40_,^[29]^ and amine‐functionalized graphene oxide nanosheets (AFGONs)^[13]^ have been reported in the literature. While many of them have their merits, some of them have a variety of drawbacks, including harsh reaction conditions, expensive catalysts, and a tedious preparation and work‐up process. Thus, the design and expansion of an alternate approach for this reaction is a priority. Given the importance of 1,4‐dihydropyridine and polyhydroquinoline, and in the framework of our previous research on the use of environment‐friendly natural polysaccharides in the synthesis of potential biologically active compounds.[^3^, ^6^, ^7^, ^30^, ^31^, ^32^] We report herein the catalytic activity investigation of BaFe_12_O_19_@GO@Fu in the synthesis of biologically active and pharmaceutically important 1,4‐dihydropyridine and polyhydroquinoline under reflux in ethanol (Figure 1b).

In addition to the catalytic investigation, the antibacterial activity of BaFe_12_O_19_@GO@Fu was evaluated on two bacterial strains, gram‐negative *Escherichia coli (E. coli)* and gram‐positive *Staphylococcus aureus* (*S. aureus*).

## 2 Results and Discussion

**Figure 2:**
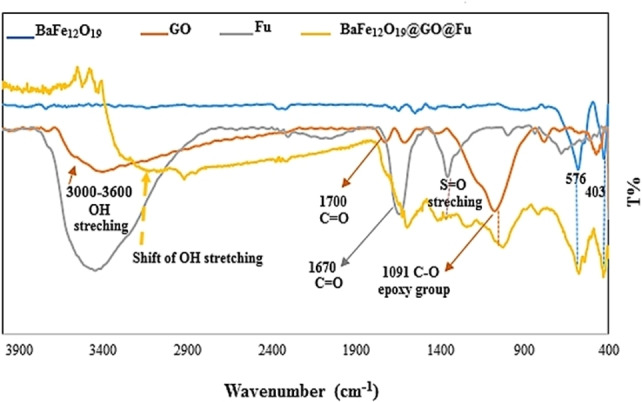
FTIR spectra of BaFe_12_O_19_, GO, Fu, BaFe_12_O_19_@GO@Fu

The FTIR spectra of BaFe_12_O_19_, GO, Fu, and BaFe_12_O_19_@GO@Fu nanocomposite are shown in Figure 2. In the FTIR spectrum of BaFe_12_O_19_@GO@Fu, the peaks at 403 and 576 cm^−1^ are related to the metal‐oxide stretching vibration from the BaFe_12_O_19_ structure. Strong broadband at 3500 cm^−1^ is associated with the stretching vibration due to the O−H of Fucoidan. A bond at 1670 cm^−1^ can be attributed to representative polysaccharide chains. The absorption band at 993 cm^−1^ indicated hemiacetal vibration at alcohol and ether functional groups in the Fucoidan structure. The peak at 1249–1431 cm^−1^ is related to the stretching vibration of S=O from the SO_3_H group. Also, in the IR spectrum of GO, the absorption band located at 3000–3600 cm^−1^ corresponds to hydrogen‐bonded O−H stretch, and the peak at 1731 cm^−1^ is related to the C=O stretching. The bending vibration of OH appears at 1614 cm^−1^ and the peak at 1091 cm^−1^ corresponds to the vibrational mode of the C−O group. The shift of OH stretching vibrations to lower wavenumber indicates the increase in intermolecular hydrogen bonding between GO and Fu. It can be seen that all the characteristic peaks of the constituents, namely GO, Fu, and BaFe_12_O_19_, are present in the IR spectrum of the nanocomposite.

**Figure 3:**
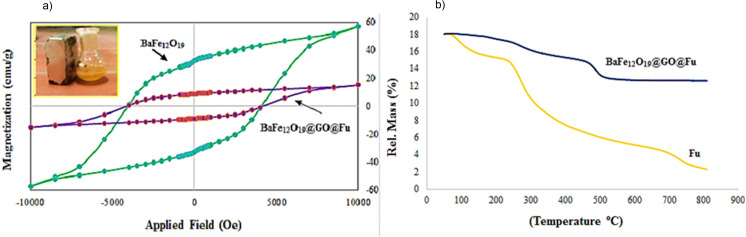
a) VSM magnetization curve of BaFe_12_O_19_, BaFe_12_O_19_@Go@Fu, and b) TGA curves of Fu and BaFe_12_O_19_@GO@Fu nanocomposite

Magnetic measurements were carried out using a vibrating sample magnetometer (VSM) at room temperature by using a magnetic field ranging from −10000 to 10000 Oe (Figure 3a). The remnant magnetization value of BaFe_12_O_19_ nanoparticles was about 57.04 emu g^−1^ implying the ferrimagnetic behavior of nanoparticles. According to the magnetization curve of the BaFe_12_O_19_@GO@Fu nanocomposite, the value of the saturation magnetization was 18.28 emu g^−1^. The smaller value of the saturation magnetization of BaFe_12_O_19_@GO@Fu compared to BaFe_12_O_19_ is due to the nonmagnetic layer‐by‐layer surface coverage.

The thermal behavior of the prepared nanocomposite was probed by TGA analyses at the range of temperature between 50 and 800 °C with the rate of 10 °C min^−1^. Figure 3b shows the TGA curves of Fucoidan and BaFe_12_O_19_@GO@Fu nanocomposite. As illustrated in the TGA curve of Fu, the majority of weight loss within a range of 200–400 °C indicated the thermal degradation of the polysaccharide. From the TGA curve of BaFe_12_O_19_@GO@Fu, it is clear that a tiny fraction of weight reduction occurred upon heating at about 100–200 °C, which relates to the elimination of volatile elements such as water from the nanocomposite. After that, the mass loss in the range of 230–335 °C to approximately 8 % and 430–500 °C just below 12 % of nanocomposite can be associated with the decomposition of GO and Fu. It should be emphasized that the BaFe_12_O_19_@GO@Fu magnetic nanocomposite shows higher thermal stability.

**Figure 4:**
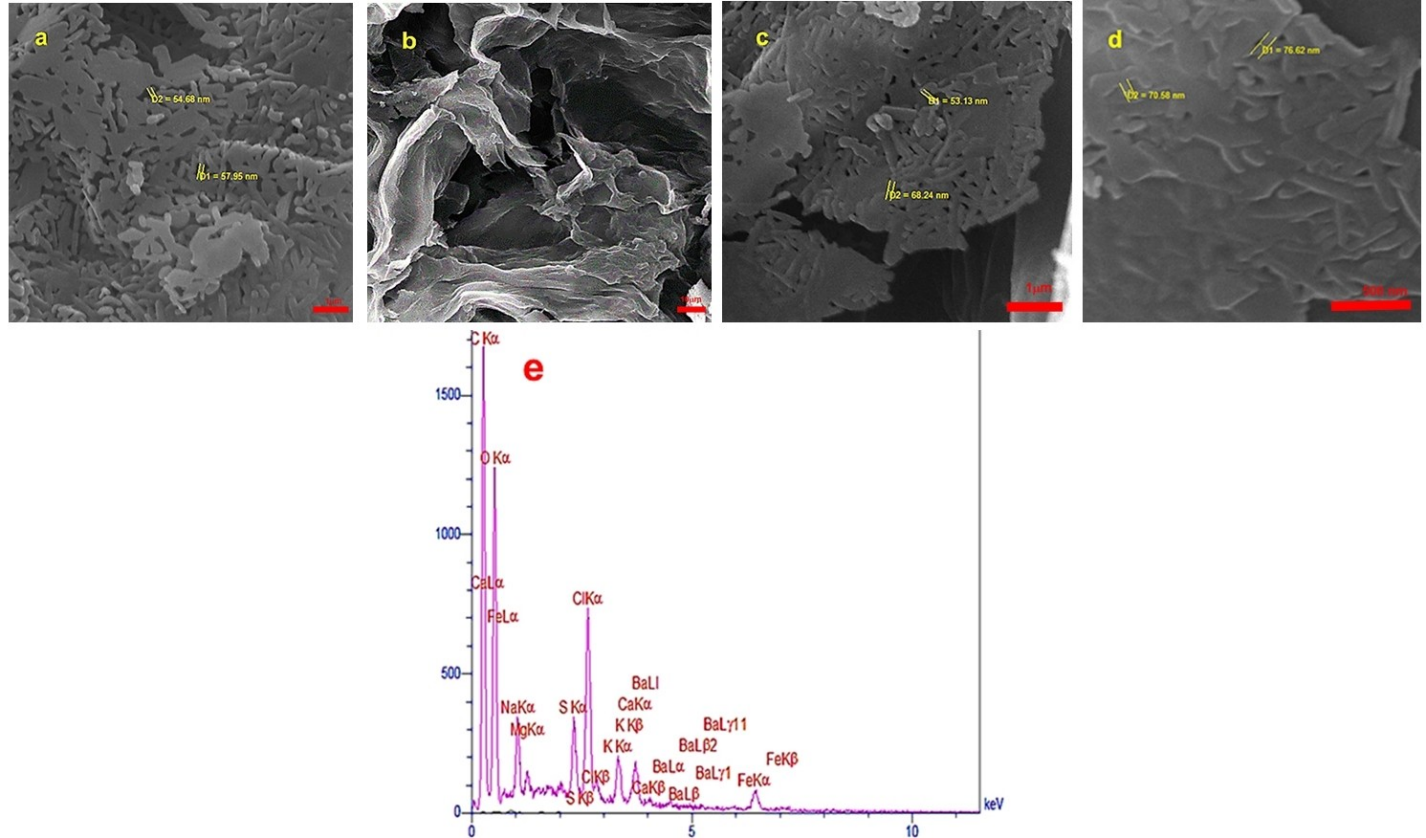
SEM images of a) BaFe_12_O_19_, b–d) BaFe_12_O_19_@GO@Fu (10 μm, 1 μm, 500 nm), and e) The EDX analysis of the BaFe_12_O_19_@GO@Fu magnetic nanocomposite.

Initially, the size, structure, and morphology of the BaFe_12_O_19_ NPs and of the BaFe_12_O_19_@GO@Fu nanocomposite were investigated by SEM analysis (Figures 4a–d), which is testimony to the fact that BaFe_12_O_19_ nanoparticles have a completely uniform M‐type structure. Furthermore, the structure and size of the nanoparticles at 10, 1 μm, and 500 nm were monitored by SEM analysis which confirms the preservation of morphology and particle size (Figures 4a–d).

In fact, the prepared nanoparticles were uniformly loaded on the composite surface and the averaged size for these nanoparticles was approximately 53–76 nm. Also, the presence of S, O, C, Ba, and Fe elements in the studied composite was confirmed by using EDX analysis, and the presence of these elements could be attributed to barium hexaferrite, graphene oxide, and fucoidan (Figure 4e).

**Figure 5:**
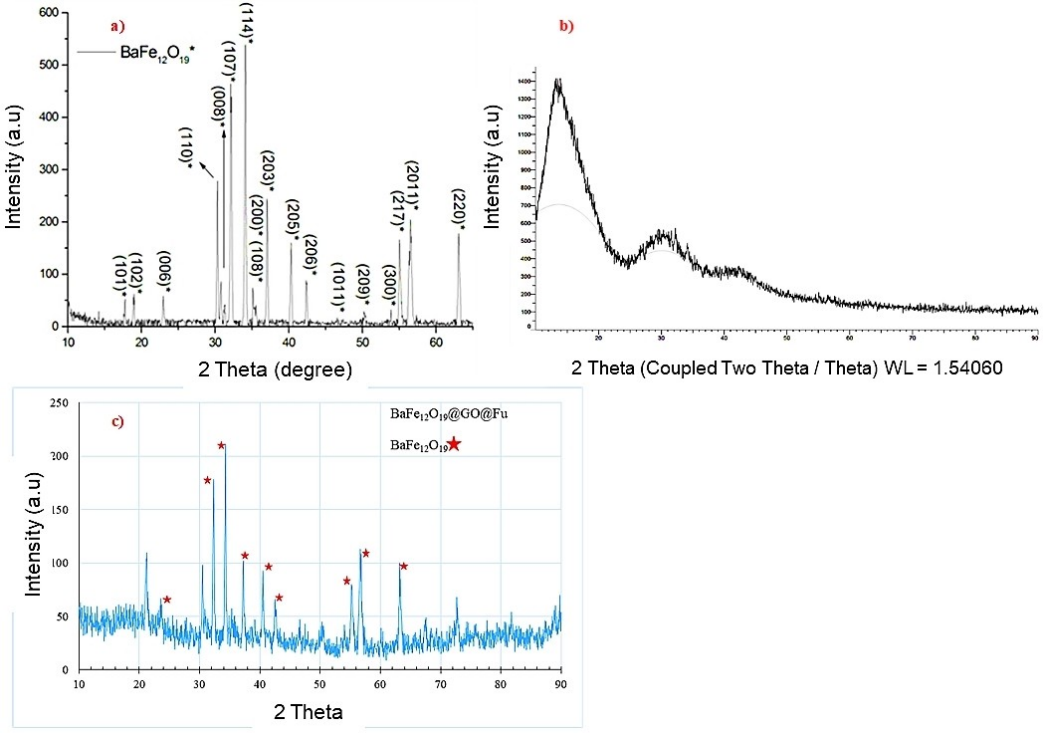
X‐ray diffraction patterns of a) the bare BaFe_12_O_19_ b) GO@Fu and c) BaFe_12_O_19_@GO@Fu.

X‐ray diffraction patterns of the bare BaFe_12_O_19_, GO@Fu, and BaFe_12_O_19_@Go@Fu are shown in Figure 5. The diffraction peaks observed at 2θ values of 29.92°, 31.68°, 33.54°, 34.84°, 36.54°, 54.61°, 56.18°, and 62.84° correspond to the crystal planes (110), (107), (114), (203), (217), (2011), (220) and confirmed that BaFe_12_O_19_ nanoparticles were synthesized in hexagonal crystal system based on the standard XRD pattern (JCPDS, card number 01–072‐0738). According to the Scherrer equation, the size of the nanoparticles amounted to 85 nm.

The XRD pattern of GO@Fu indicates a sharp peak at about 2θ≈12° which is related to graphene oxide and another peak at about 2θ≈27° which can refer to the amorphous nature of Fu (Figure 5b). The XRD pattern of BaFe_12_O_19_@GO@Fu nanocomposite indicates that the crystal structure of barium hexaferrite has been conserved after modification, and at the same time, the existence of a small bump in 2θ about 12° and 27° might confirm the presence of GO and Fu in the nanocomposite (Figure 5c).

**Figure 6:**
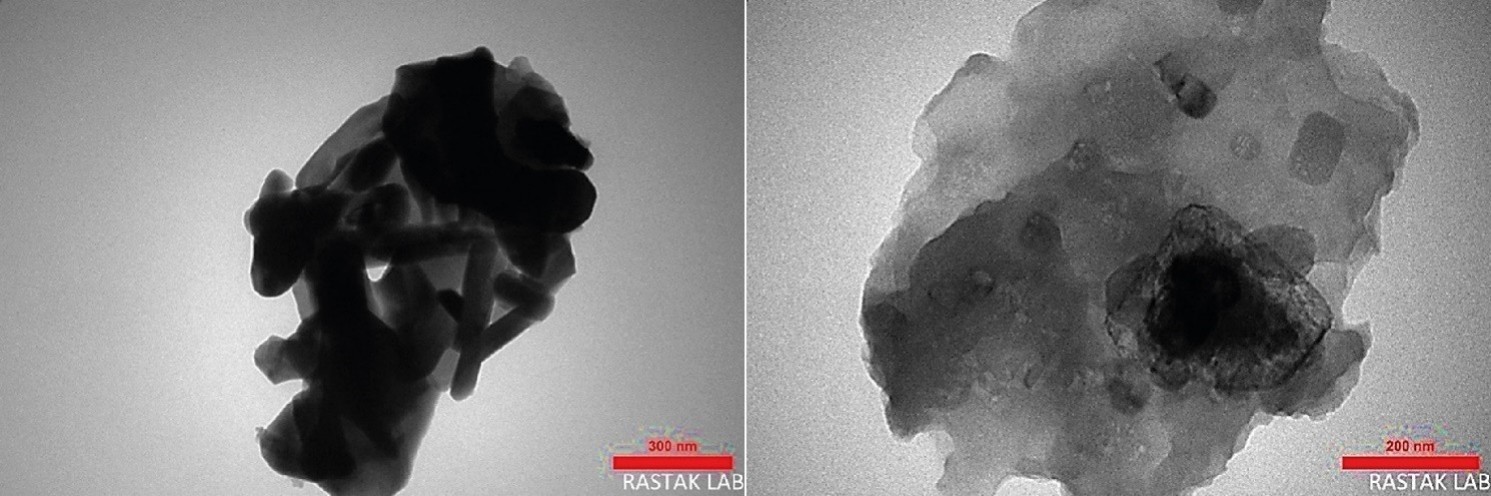
TEM images of BaFe_12_O_19_@GO@Fu nanocomposites at different magnification.

The shape and size of the nanoparticles BaFe_12_O_19_@Go@Fu were examined by Transmission Electron Microscopy (TEM) analysis. TEM images with different magnifications (300 and 200) reveal the layered nature of the composites and a random distribution of the rod‐shaped BaFe_12_O_19_ nanoparticles (black parts) in the GO sheets (white parts) which approves the interactions between nanoparticles and GO surface (Figure 6).

**Figure 7:**
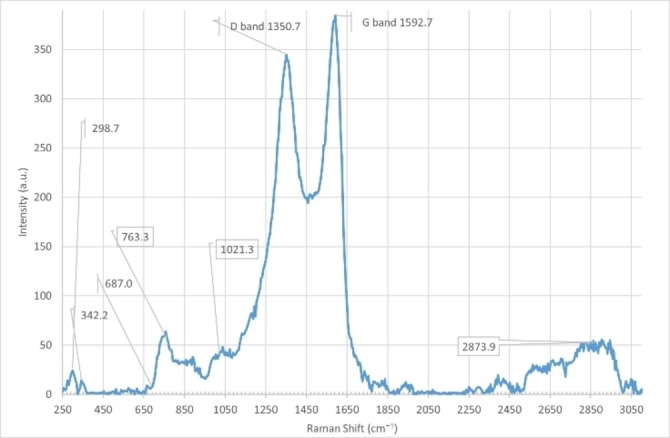
Raman spectrum of BaFe_12_O_19_@GO@Fu.

The BaFe_12_O_19_@GO@Fu nanocomposite was characterized by Raman spectroscopy analysis as shown in Figure 7. The D band of GO is found at 1351 cm^−1^ which is associated with the disorder stemming from oxygen moieties and the G band is found at 1593 cm^−1^ due to C−C stretching. The observed intensity ratio (*I*_D_/*I*_G_) was 0.87 for multilayer GO. The Raman spectrum also shows some characteristic peaks of the M‐type barium ferrite phase. The peaks at 758 and 684 cm^−1^ can be assigned to A_1g_ vibrations of Fe−O bonds at the tetrahedral 4_f1_ and bipyramidal 2_b_ sites, respectively.^[34]^ The peaks at 299 cm^−1^ are due to E_1g_ vibrations, while the peak at 342 cm^−1^ is due to E_2g_ vibration. The Raman band at 840 cm^−1^ is attributed to the COS bending vibration of the sulfate group in fucoidan.

### 2.1 Antibacterial activity of BaFe_12_O_19_@GO@Fu

**Figure 8:**
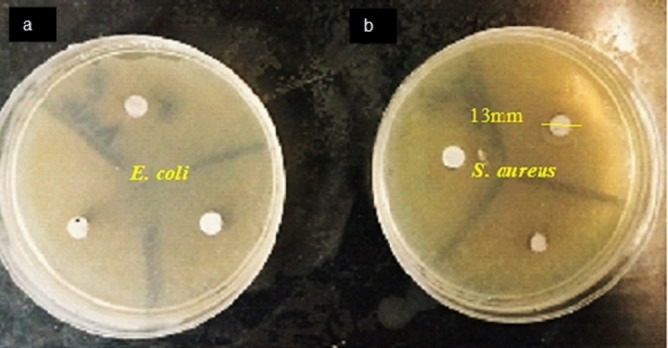
Antibacterial activity of BaFe_12_O_19_@GO@Fu against a) *E. coli* and n) S*. aureus*.

The antibacterial efficacy of BaFe_12_O_19_@GO@Fu was investigated by the Agar well diffusion method (Figure 8). A suspension of bacteria (*E. coli* or *S. aureus*) was diffused on the agar plates of Mueller‐Hinton (MH) supplemented with Tween 80 surfactants (final concentration of 0.05 % v/v) using a final density of 1.5×108 colony‐forming units (CFU)/ml of tested bacterial strains suspended in MH. Subsequently, on the surface of the previously inoculated agar plate, 50 mg of sample was placed which was firstly kept at 4 °C for 2 h, then incubated at 37 °C for 24 h. The clear zones of inhibition (13 mm) around the antimicrobial sample revealed that BaFe_12_O_19_@GO@Fu has antibacterial activity against gram‐positive bacteria *S. aureus*.

In connection with the mechanism of this inhibitn, studies to date have not yet determined the exact mechanism for antibacterial activity, but the possible mechanism could be based on the binding of sulfate groups in fucoidan to the bacterial cell wall resulting in bacterial cell membrane destruction, and leakage of intracellular material to the outside, eventually causing the death of the bacteria.

### 2.2 Catalytic Activity of BaFe_12_O_19_@GO@Fu Magnetic Nanocomposite

The catalytic efficacy of BaFe_12_O_19_@GO@Fu was investigated in the one‐pot reaction between 4‐chlorobenzaldehyde (1 mmol), ethyl acetoacetate (1 mmol), dimedone (1 mmol), and ammonium acetate (2 mmol) as a model reaction for the synthesis of polyhydroquinoline derivatives. In the case of 1,4‐dihydropyridines, ethyl acetoacetate (2 mmol) has been used instead of dimedone. Both of the model reactions were performed in the presence of different solvents such as ethanol, water, toluene, dichloromethane, acetonitrile, and also under solvent‐free conditions. The results show a substantial increase in the yield of the reaction when ethanol was used as a solvent. To find the optimum catalyst loading, the model reactions were studied, regarding various amount of catalyst (Table 1). Although the steady increase in the amount of catalyst from 5 to 15 mg (entries 7 to 10) gives noticeable yield, a further quantity of catalyst does not give significant change in the yield of the reaction. Model reactions were carried out in the absence of catalyst (entry 1) and with Fu, BaFe_12_O_19_, GO@Fu, and BaFe_12_O_19_@GO@Fu (entries 2 to 5). These results endorsed that BaFe_12_O_19_@GO@Fu was more appropriate for these reactions. Overall, the most influential conditions for the desired products were found to be refluxing in ethanol in the presence of 15 mg magnetic nanocomposite.

**Table 1:** Optimization of the catalyst, temperature, and the solvent for the synthesis of 1,4‐dihydropyridine and polyhydroquinolines.

Product 5d^[b]^		Product 6 m^[a]^		Solvent	Temperature	Catalyst (amount in [mg])	Entry
Yield (%)	Time (min)	Yield (%)	Time (min)				
**trace**	300	trace	300	Solvent free	r.t.	–	1
**78**	20	80	20	Ethanol	Reflux	GO (15)	2
**61**	20	65	20	Ethanol	Reflux	BaFe_12_O_19_ (15)	3
**80**	20	85	20	Ethanol	Reflux	Fu (15)	4
**85**	15	89	10	Solvent free	r.t.	BaFe_12_O_19_@GO@Fu (15)	5
**90**	15	91	10	Ethanol	r.t.	BaFe_12_O_19_@GO@Fu (15)	6
**90**	15	90	12	Ethanol	Reflux	BaFe_12_O_19_@GO@Fu (5)	7
**91**	15	93	12	Ethanol	Reflux	BaFe_12_O_19_@GO@Fu (10)	8
**95**	15	95	12	Ethanol	Reflux	BaFe_12_O_19_@GO@Fu (12)	9
**96**	15	97	12	Ethanol	Reflux	BaFe_12_O_19_@GO@Fu (15)	10
**90**	15	91	12	Ethanol	Reflux	BaFe_12_O_19_@GO@Fu (20)	11
**78**	20	80	20	H_2_O	Reflux	BaFe_12_O_19_@GO@Fu (15)	12
**90**	15	91	15	Ethanol/H_2_O	Reflux	BaFe_12_O_19_@GO@Fu (15)	13
**60**	30	60	30	CH_3_CN	Reflux	BaFe_12_O_19_@GO@Fu (15)	14
**55**	35	60	35	Toluene	Reflux	BaFe_12_O_19_@GO@Fu (15)	15
**50**	45	55	45	CH_2_Cl_2_	Reflux	BaFe_12_O_19_@GO@Fu (15)	16

[a] Reaction conditions: 4‐chlorobenzaldehyde (1 mmol), ethyl acetoacetate (1 mmol), dimedone (1 mmol), ammonium acetate (1 mmol), and 3 ml solvent; [b] reaction conditions: 4‐chlorobenzaldehyde (1 mmol), ethyl acetoacetate (2 mmol), ammonium acetate (2 mmol), and 3 ml solvent.

For overall evaluation of the synthesis of 1,4‐dihydropyridines and polyhydroquinolines after the aforementioned optimization of conditions, a range of various aromatic aldehydes were chosen. As can be seen in Table 2, substrates with both electron‐donating and electron‐withdrawing substituents were investigated in these reactions. The presence of electron‐withdrawing substituents tended to increase the reaction rate, and the electron‐donating group slowed down the process. The results clearly show that the reaction of diverse aromatic aldehydes, ammonium acetate, ethyl acetoacetate, and dimedone under reflux with the presence of the nanocomposite provided the corresponding products in high yields (90–96 %) at appropriate reaction times. As most of the products crystallized directly from the reaction mixture, all of the products were characterized by their melting points, and some of the products were additionally characterized by NMR spectral data (s. Supporting Information).

**Table 2:** Synthesis of 1,4‐dihydropyridine and polyhydroquinolines catalyzed by BaFe_12_O_19_@GO@Fu nanocomposite.

Entry	Product	Aldehyde	Time [min]	Yield [%]	M.p. [°C] Found/Reported
**1**	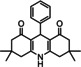	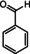	15	90	286–288/285–289^[36]^
	5a				
**2**	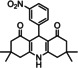	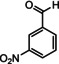	12	92	292–295/ >220^[37]^
	5b				
**3**	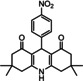	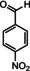	10	94	289–290/290–292^[38]^
	5c				
**4**	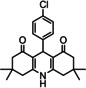	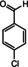	12	95	298–300/ >220^[36]^
	5d				
**5**	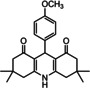	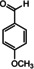	15	92	275–278/274–276^[3]^
	5e				
**6**	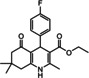	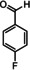	15	94	183–185/182–184^[39]^
	6f				
**7**	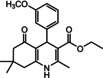	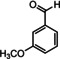	16	92	202–204202‐204^[40]^
	6 g				
**8**	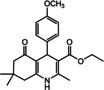	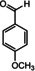	14	93	255–257/255–257^[41]^
	6 h				
**9**	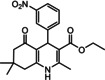	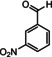	10	96	179–181/180–183^[29]^
	6i				
**10**	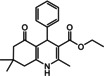	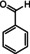	12	92	203–204/203–205^[28]^
	6j				
**11**	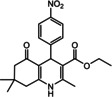	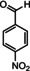	14	95	238–241/235–240^[29]^
	6k				
**12**	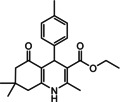	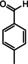	15	90	254–256/255–257^[29]^
	6 l				
**13**	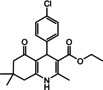	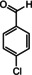	15	96	245–246/244–246^[41]^
	6 m				
**14**	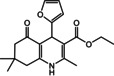	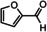	14	92	225–228/226–228^[42]^
	6n

To reveal the effectiveness of the bio‐based BaFe_12_O_19_@GO@Fu nanocatalyst, a comparison was made with some catalysts previously used in the synthesis of 1,4‐dihydropyridine. As indicated in Table 3, compared to other catalysts, BaFe_12_O_19_@GO@Fu represented an environmental condition with excellent TON and TOF (6330 and 25320 h^−1^).

**Table 3:** Comparison of the catalytic activity of BaFe_12_O_19_@GO@Fu in the synthesis of 1,4‐dihydropyridine (**5 d**) with some other catalysts reported in the literature.

Entry	Catalyst (amount in^[^mg])	Conditions	Time [min]	Yield^[^%]	Ref.	TON/TOF^[^h^−1^]
**1**	Fe_3_O_4_−TiO_2_−SO_3_H (n‐FTSA)^[a]^ (10)	EtOH/Reflux	50	95	[43]	9500/11400
**2**	AFGONs^[b]^ (25)	EtOH/ r.t.	180	89	[13]	3560/1187
**3**	Cell‐Pr−NHSO_3_H (50)	EtOH/Reflux	42	90	[44]	1800/2571
**4**	MgAl_2_−HT^[c]^ (50)	CH_3_CN/ r.t.	390	53	[45]	1060/163
**5**	BaFe_12_O_19_@GO@Fu (15)	EtOH/Reflux	15	95	**This work**	6330/25320

[a] nano‐Fe_3_O_4_−TiO_2_−SO_3_H ;[b] Amine‐functionalized graphene oxide nanosheets;[c] MgAl2 prepared by hydrothermal method.

**Figure 9:**
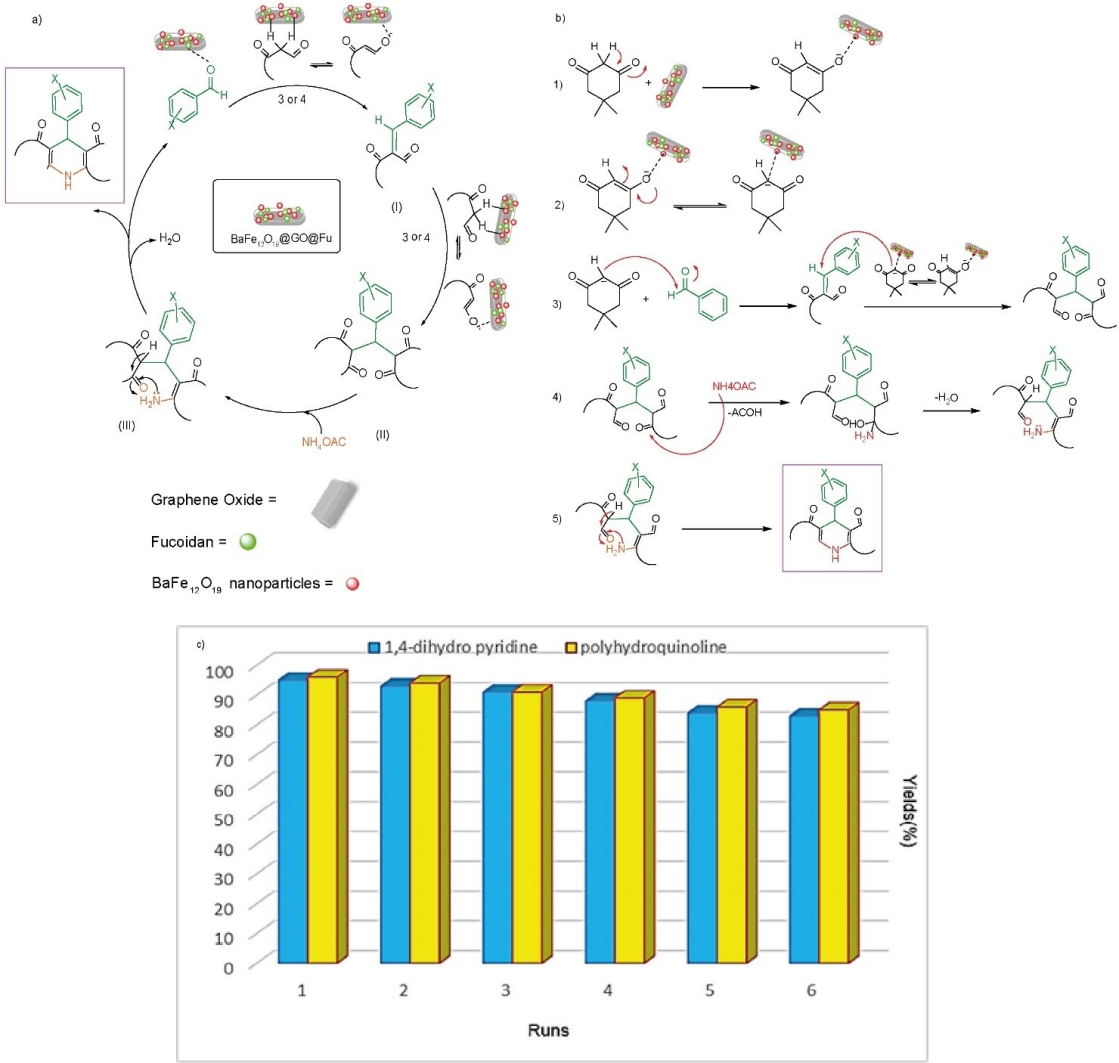
a) Proposed mechanism for the synthesis of 1,4‐dihydropyridine and polyhydroquinoline derivatives by using BaFe_12_O_19_@GO@Fu nanocomposite; b) electron transfer paths of the reaction and c) recycling study of BaFe_12_O_19_@GO@Fu in the model reactions for the syntheses of **5 d** and **6 l**.

A plausible mechanism for the formation of 1,4‐dihydropyridine and polyhydroquinoline derivatives from one‐pot reactions of an aromatic aldehyde, dimedone or ethyl acetoacetate, and ammonium acetate in the presence of BaFe_12_O_19_@GO@Fu nanocomposite as a bifunctional catalyst is illustrated in Figure 9a. The surface of BaFe_12_O_19_ contains Ba^2+[35]^ which can act as Lewis acid and the negatively charged Fu can act as Lewis base. Therefore, BaFe_12_O_19_@GO@Fu could activate the reactants and moreover, due to the hygroscopic character of fucoidan, would adsorb the water formed during the reaction and subsequently favor the forward reaction. Initially, intermediate (I) is formed from the condensation reaction between an aromatic aldehyde and dimedone **3** or ethyl acetoacetate **4** with nanocomposite. Next, the second equivalent of **3** or **4** reacts with intermediate (I), producing intermediate (II). Ammonia released from the decomposition of ammonium acetate, then attacks the activated carbonyl. Then, the cyclic intermediate (III) is formed and H_2_O is produced as a byproduct. At the end of this processes an imine‐enamine tautomerization forms the final products (**5 a**–**e** and **6 f**–s**m**). The reaction mechanism and electron transfer pathways for each step were expanded in Figure 9b.

## 3 Conclusions

In summary, we have presented the synthesis and characterization of a new polymer‐coated magnetic nanocomposite based on graphene oxide, marine sulfated polysaccharide fucoidan, and barium ferrite, BaFe_12_O_19_@GO@Fu. The first successful catalytic application of BaFe_12_O_19_@GO@Fu was examined in two fundamental multicomponent reactions for the preparation of 1,4‐dihydropyridines and polyhydroquinolines. It should be noted that due to the synergy that exists between fucoidan, graphene oxide, and magnetic ferrite, BaFe_12_O_19_@GO@Fu can be considered an environmentally friendly nanocomposite, exhibiting excellent bifunctional catalytic activity and stability, alongside cost‐effectiveness, non‐toxicity, simple work‐up procedure, high yields, and reusability. Simultaneously, potential antibacterial activity for BaFe_12_O_19_@GO@Fu was observed against *S. aureus*.

## Experimental Section

### Materials and Methods

All solvent, chemicals were purchased by Merck, Aldrich chemical companies and used without further purification. Melting points were measured on an Electrothermal 9100 apparatus. Synthesis of catalyst was sonicated using Elma at 60 Hz. The FTIR Spectra were recorded as KBr pellets through Shimadzu IR‐470 spectrophotometer. Thermogravimetric analysis (TGA) was measured by Bahr‐STA 504 instrument. The SEM images were carried out using a TESCAN instrument. Elemental analysis of the nanocomposite was done by EDX analysis recorded by TESCAN4992. NMR spectra were recorded on Varian – INOVA 500 MHz spectrometer. X‐ray diffraction (XRD) pattern was obtained on a D8‐Advance Bruker. Magnetic measurements were achieved by using VSM model MDKFD from Danesh Pajohan Kavir Co. Kashan.

### Synthesis of Graphene Oxide

According to Hummer^’^s method, graphite (1 g) was added into a round bottom flask containing 1 g of sodium nitrate and 23 ml of concentrated H_2_SO_4_. The obtained mixture was sonicated for 30 min, then, by adding ice to the mixture, the temperature was decreased below 10 °C. Next, 3 g of KmnO_4_ was gradually added to the mixture. The temperature was increased up to 35 °C . Afterwards, a brown suspension was obtained by adding slowly 50 ml of water from 35 °C to 98 °C. H_2_O_2_ (30 %) and 2 ml of HCl (32 %) were added to the diluted solution, respectively‐ Finally, the obtained sediment was washed with DI water several times to remove all the acids.

### Synthesis of Barium Hexaferrite (BaFe_12_O_19_) Magnetic Nanoparticles

Barium hexaferrite, BaFe_12_O_19_, was synthesized according to our previously published procedure through the co‐precipitation‐calcination procedure.^[33]^ Co‐precipitation was conducted using an aqueous mixture of barium nitrate (Ba(NO_**3**_)_2_, 1.99 mmol), iron(III) nitrate nonahydrate (Fe(NO_3_)_3_ ⋅ 9 H_2_O, 23.76 mmol) and citric acid (4.5 g, 23.42 mmol) using aqueous ammonia solution (25 %) at pH 8 and room temperature. The solid was filtered, washed, and dried at 80 °C. The obtained BaFe_12_O_19_ was calcined in N_2_ atmosphere at 750 °C for 2 h to achieve the hexagonal structure of magnetic BaFe_12_O_19_ nano‐powder.

### Synthesis of BaFe_12_O_19_@GO@Fu Nanocomposites

**Figure 10:**
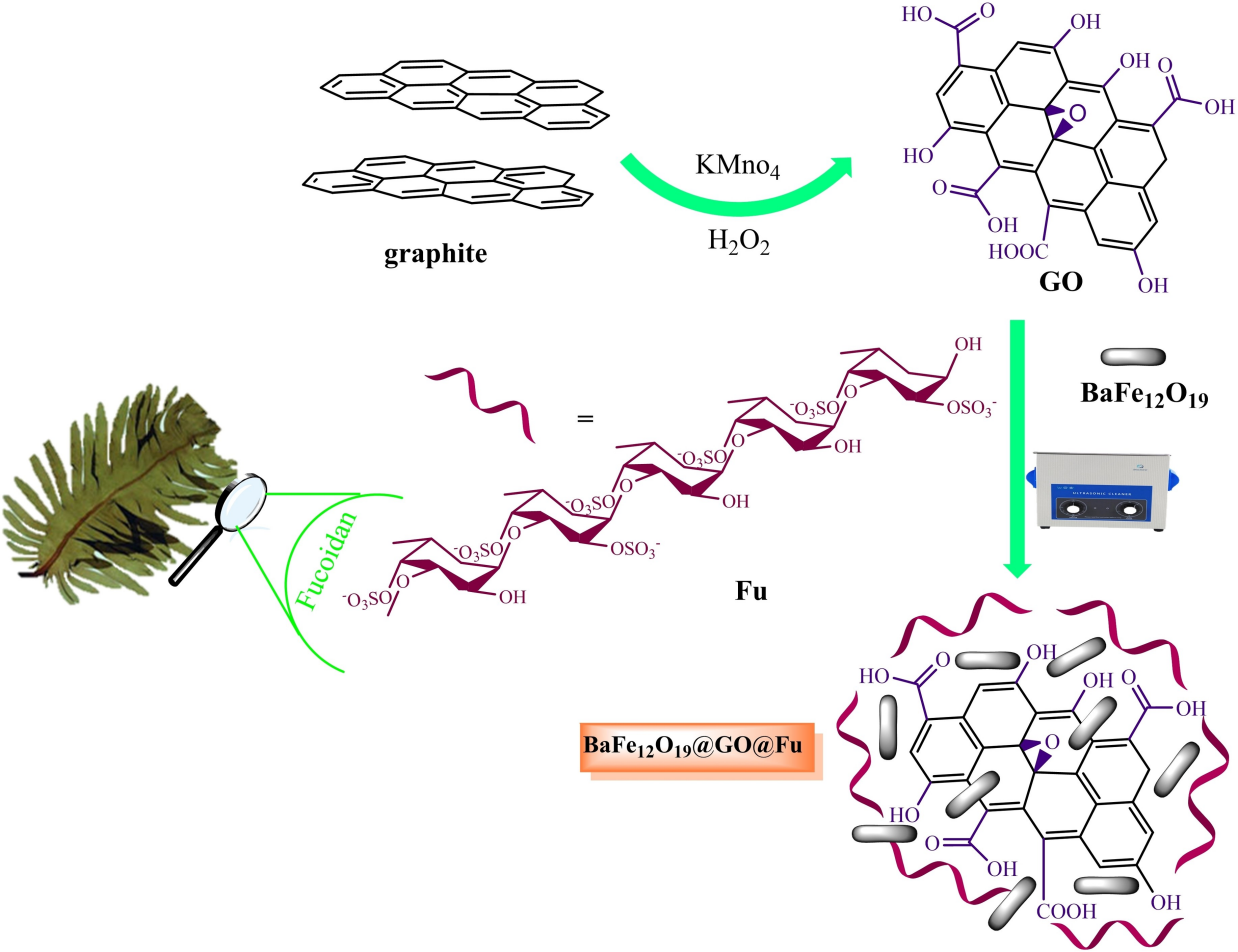
Schematic preparation of BaFe_12_O_19_@GO@Fu magnetic nanocomposite.

Initially, GO (0.3 g) was dispersed in deionized (DI) water. Then 1.5 g of fucoidan algae was ball‐milled (5 min) and added to the reaction mixture. Subsequently, an aqueous solution of NaOH (0.1 m) was gradually added until reaching pH=10. The reaction mixture was refluxed at 95 °C for 3 h before adding BaFe_12_O_19_ (0.3 g, 0.27 mmol). Finally, this mixture was dispersed by ultrasonication at 60 °C for 1 h. The prepared nanocomposite was separated, washed with EtOH and DI water, and dried in an oven at 60 °C (Figure 10).

### General Procedure for the Synthesis of 1,4‐Dihydropyridine Derivatives

A mixture of ethyl acetoacetate (2 mmol), ammonium acetate (2 mmol), and aldehydes (1 mmol) in the presence of BaFe_12_O_19_@GO@Fu nanocomposite (0.015 g) was vigorously stirred in a round bottom flask under reflux in ethanol (3 ml). The progression of the reaction was checked by thin‐layer chromatography (TLC; ethyl acetate:*n*‐hexane 1 : 3 v/v). After the reaction was finished, the catalyst was separated simply by an external magnet, and the products were separated by filtration, washed with EtOH, DI water and recrystallized to yield pure products.

### General Procedure for the Synthesis of Polyhydroquinoline Derivatives

In a round‐bottomed flask equipped with a reflux condenser and magnetic stirrer, a mixture of aromatic aldehydes (1 mmol), ethyl acetoacetate (1.0 mmol), dimedone (1 mmol), ammonium acetate (2 mmol), and a catalytic amount of BaFe_12_O_19_@GO@Fu was stirred under reflux in ethanol for an appropriate time. The advancement of the reaction was monitored by TLC. After completion of the reaction, the magnetic BaFe_12_O_19_@GO@Fu was separated using a bar magnet. The purification of the crude product was achieved by recrystallization from EtOH, and the pure product was dried in an oven. The magnetic BaFe_12_O_19_@GO@Fu was washed consecutively by EtOH and deionized water, then dried at 60 °C before being reused in the next run of reactions.

## Supporting Information

^1^H NMR and ^13^C NMR spectral data of selected products are shown in the Supporting Information file.

## Conflict of interest

The authors declare no conflict of interest.

4

## Supporting information

As a service to our authors and readers, this journal provides supporting information supplied by the authors. Such materials are peer reviewed and may be re‐organized for online delivery, but are not copy‐edited or typeset. Technical support issues arising from supporting information (other than missing files) should be addressed to the authors.

Supporting Information

## Data Availability

The data that support the findings of this study are available in the supplementary material of this article.
