# Supplementary material for: Preparation, Antibacterial Activity, and Catalytic Application of Magnetic Graphene Oxide‐Fucoidan in the Synthesis of 1,4‐Dihydropyridines and Polyhydroquinolines
Source: ChemistryOpen. 2021 Dec 1;10(12):1186–96. doi: 10.1002/open.202100221 (PMC8634770; doi:10.1002/open.202100221)
Supplement: Supplementary file 1 — Supporting Information [file OPEN-10-1186-s001.pdf]

ChemistryOpen

Supporting Information

Preparation, Antibacterial Activity, and Catalytic Application of Magnetic Graphene Oxide-Fucoidan in the Synthesis of 1,4-Dihydropyridines and Polyhydroquinolines

Aliakbar Nosrati, Sara Amirnejat, and Shahrzad Javanshir*

Supporting Information

Spectral ^{1}HNMR data of the selected products

Ethyl 4-(4-chlorophenyl)-2,7,7-trimethyl-5-oxo-1,4,5,6,7,8-hexahydroquinoline-3-carboxylate (6m)

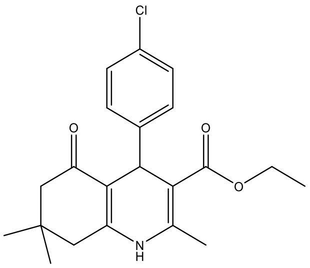

M.p.: 245 °C. IR: (KBr, cm^{-1}): 3273, 3192, 1703, 1600, 1486, 1377, 1278, 1208, 1153, 842. ^{1}H NMR (500 MHz, DMSO): δH (ppm) = 0.82 (s, 3H, CH3), 1.0 (s, 3H, CH3), 1.1 (t, 3H, J=5Hz, CH_3), 1.97(d-d, 2H, CH2), 2.16 (d-d, 2H, CH_2), 2.25 (s, 3H, CH_3), 3.95 (q, 2H, J=10 Hz CH_2), 4.82 (s, 1H, CH), 7.14(d, 2H, J= 10Hz, H-Ar), 7.23 (d, 2H,J=10Hz, H-Ar), 9.09 (s, 1H, NH). ^{13}C NMR (125 MHz, DMSO): δ (ppm) = 14.6, 18.7, 26.9, 29.5, 32.6, 36.04, 51.6, 59.6, 103.6, 110.09, 128.1, 129.7, 130.6, 145.8, 147.0, 150.05, 167.1, 194.7.

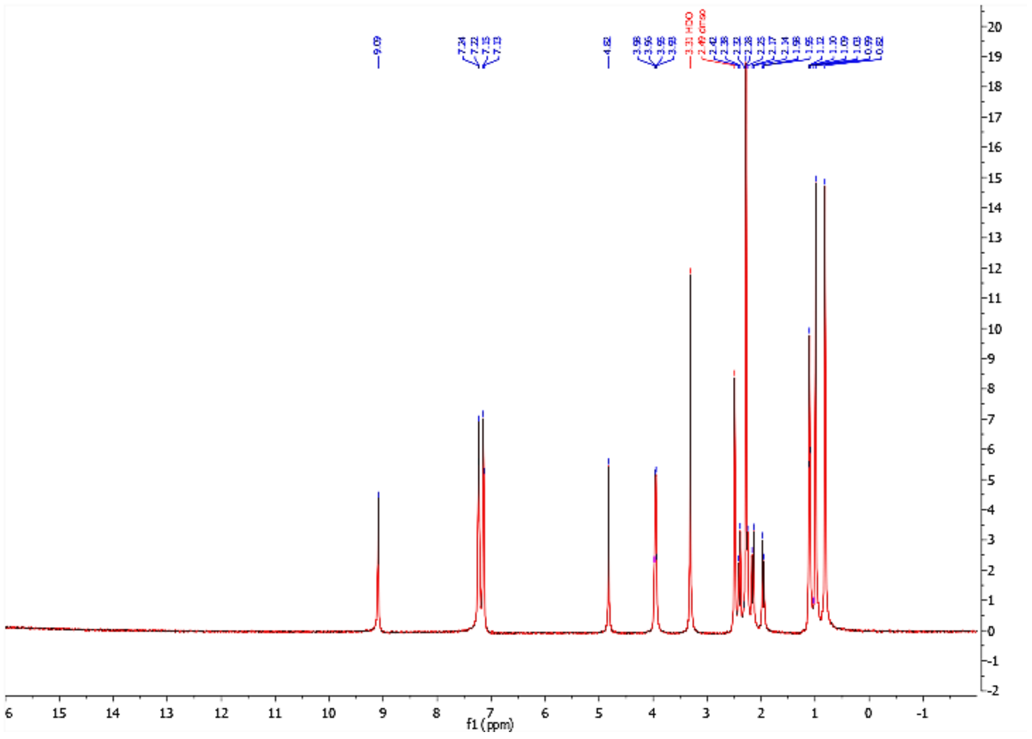

^{1}HNMR of ethyl 4-(4-chlorophenyl)-2,7,7-trimethyl-5-oxo-1,4,5,6,7,8-hexahydroquinoline-3-carboxylate (**6m**)

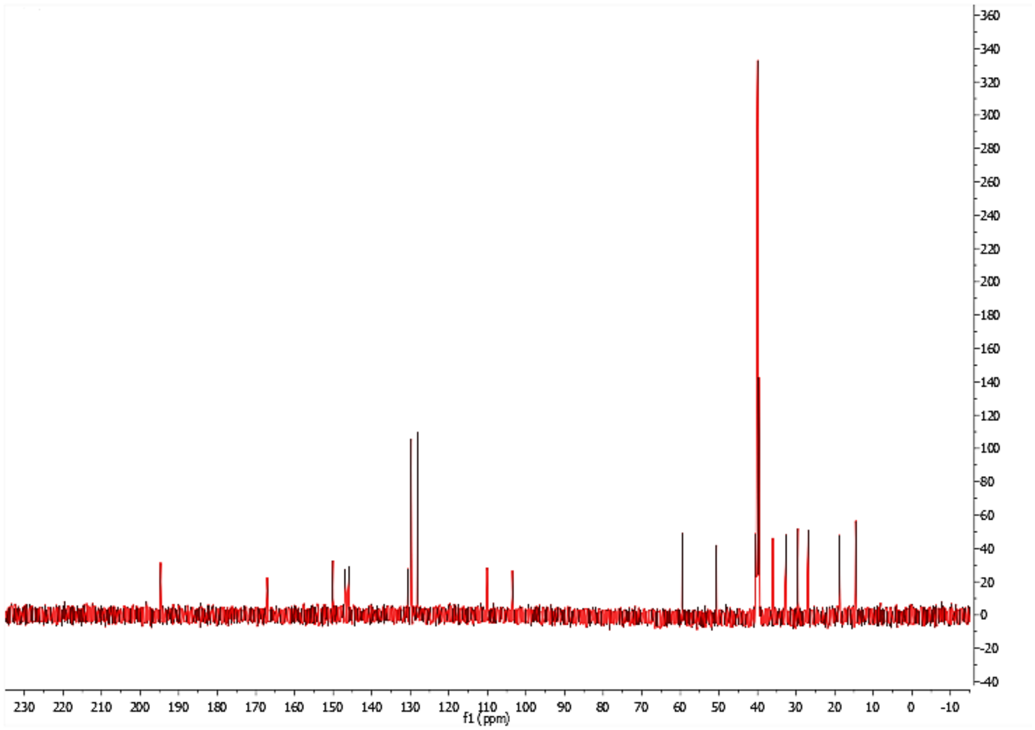

[1]CNMR of ethyl 4-(4-chlorophenyl)-2,7,7-trimethyl-5-oxo-1,4,5,6,7,8-hexahydroquinoline-3-carboxylate (**6m**)

9-(4-chlorophenyl)-3,3,6,6-tetramethyl-3,4,6,7,9,10-hexahydroacridine-1,8(2*H*,5*H*)-dione (5d)

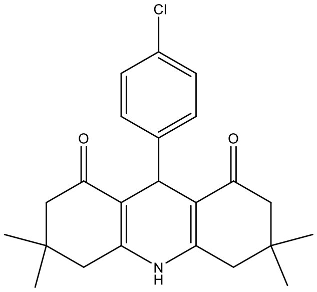

M.p.: 299 °C. IR (KBr, cm^{-1}): 3276, 3197, 2956, 1664, 1608, 1485, 1368, 1243, 1148. ^{1}H NMR (500 MHz, DMSO): δH (ppm) = 0.85 (s, 6H, CH3), 1.0 (s, 6H, CH3), 1.98-2.49 (m, 8H, CH_2) 4.77 (s, 1H, CH), 7.15–7.22 (m, 4H, H-Ar), 9.37 (s, 1H, NH). ^{13}C NMR (125 MHz, DMSO): δ (ppm) = 26.89, 29.5, 32.6, 33.15, 50.57, 111.48, 127.99, 129.9, 130.39, 146.5, 149.96, 194.8.

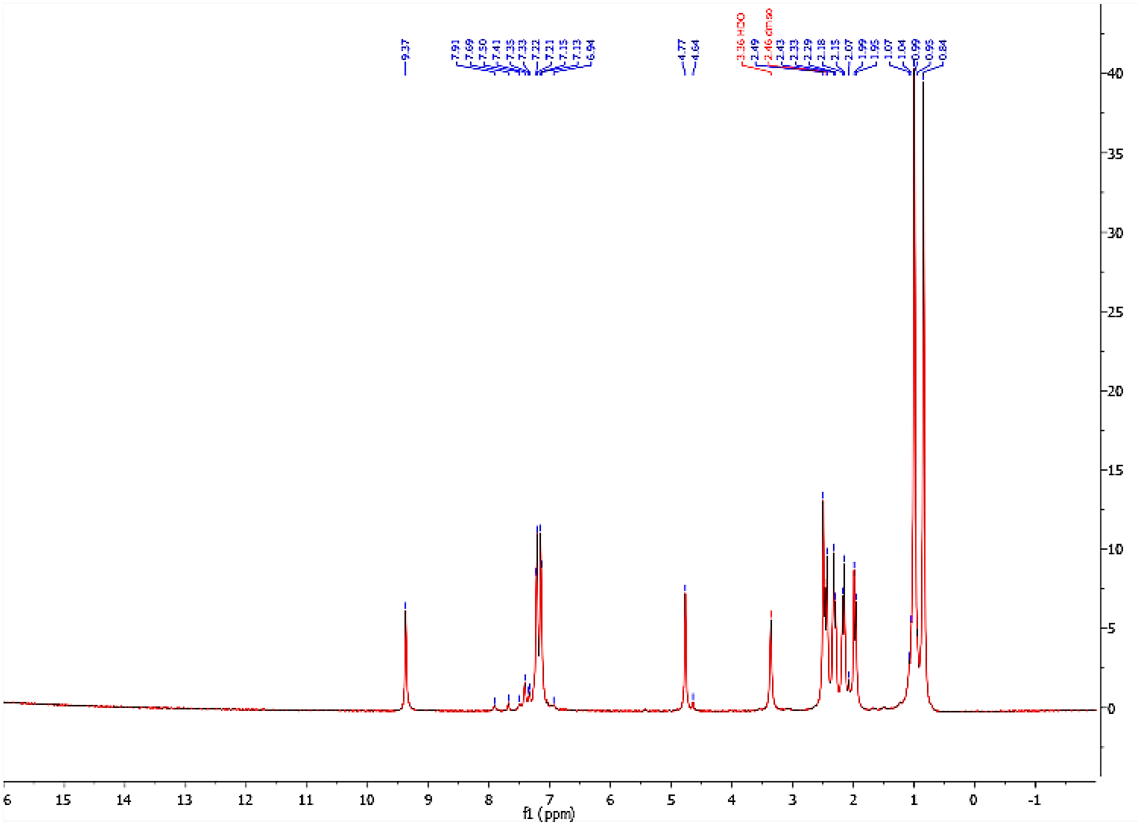

1HNMR of 9-(4-chlorophenyl)-3,3,6,6-tetramethyl-3,4,6,7,9,10-hexahydroacridine-1,8(2*H*,5*H*)-dione (**5d**)

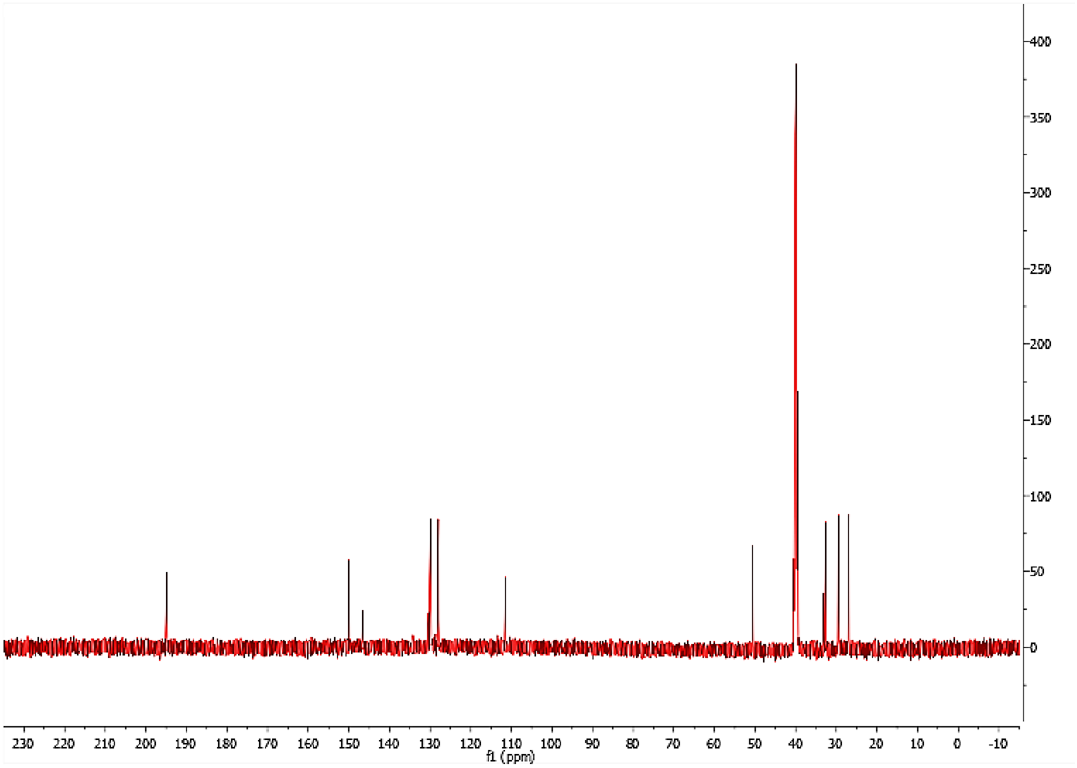

^{13}CNMR of 9-(4-chlorophenyl)-3,3,6,6-tetramethyl-3,4,6,7,9,10-hexahydroacridine-1,8(2*H*,5*H*)-dione (**5d**)
